# Comparison of thermal, rheological properties of Finnish *Pinus* sp. and Brazilian *Eucalyptus* sp. black liquors and their impact on recovery units

**DOI:** 10.1038/s41598-024-66513-z

**Published:** 2024-07-05

**Authors:** Jesús Nuncira, Getúlio Francisco Manoel, Larisse Ap. Ribas Batalha, Lindomar Matias Gonçalves, Clara Mendoza-Martinez, Marcelo Cardoso, Esa K. Vakkilainen

**Affiliations:** 1 LUT University, Yliopistonkatu 34, 53850 Lappeenranta, Finland; 2 Pontifical Catholic University of Minas Gerais (PUC Minas), Belo Horizonte, MG 30535-000 Brazil; 3 Federal Rural University of Rio de Janeiro (UFRRJ), Seropédica, RJ 23890-000 Brazil; 4 Institute of Pure and Applied Sciences, Federal University of Itajubá (UNIFEI), Rua Irmã Ivone Drumond, 200 - Industrial District II, Itabira, MG 35903-087 Brazil; 5 Federal University of Minas Gerais (UFMG), Belo Horizonte, MG 31270-901 Brazil

**Keywords:** Apparent viscosity, Dynamic shear properties, *Pinus* sp. and *Eucalyptus* sp. black liquors, Recovery boiler, Thermal properties, Energy science and technology, Engineering, Materials science

## Abstract

Black liquor (BL) is the major bioproduct and biomass fuel in pulp mill processes. However, the high viscosity of BL makes it a challenging material to work with, resulting in issues with evaporators and heat exchangers during its transport and processing. The thermal and rheological properties of BLs from *Pinus* sp. (PBL) and *Eucalyptus* sp. (EBL) were studied. FTIR spectra revealed the presence of the characteristic functional groups and the chemical composition in liquors. TGA/DTG curves showed three characteristic degradation stages related to evaporation of water, pyrolysis of organic groups, and condensation of char. Rheologically, liquors are classified as non-Newtonian and with comportment pseudoplastic. Their rheological dynamic shear properties included a linear viscoelastic region up to 1% shear strain, while frequency sweeps showed that storage modulus (Gʹ) > loss modulus (Gʹʹ), thus confirming the solid-like behavior of both BLs. The rheological study demonstrated that increasing the temperature and oscillatory deformations of PBL and EBL decreased their degree of viscoelasticity, which could favor their pumping and handling within the pulp mill, as well as the droplet formation and swelling characteristics in the recovery furnace.

## Introduction

Worldwide, the pulp and paper industry (PPI) produces around 1.3 billion tons of weak black liquor (wBL) per year and 200 million tons of strong black liquor (sBL), which is used as combustion fuel in the recovery boiler. In the same process, about 50 million tons of inorganic cooking chemicals are recovered, and roughly 700 million tons of high-pressure steam are produced^1^. Currently, black liquor (BL) is the fifth most important fuel in the world after to coal, oil, natural gas, and gasoline. In Finland and Sweden, where significant amounts of pulp and paper are produced, BL is the primary renewable biofuel^2^. Less energy is consumed for steam production with BL (13,000–15,000 kJ kg^−1^) than for fossil fuels conversion processes, due to the amount of heat used for the water evaporation, the production of Na_2_S, and the heat conducted with the smelt. Between 2.5 and 3.8 kg of steam is generated per kg of BL dry solids (ds). Depending on the quality of the steam and the type of turbine, a kraft pulp mill of 1000 tons generates up to 35 MW of power per day by burning 1500 tons of BL per day^2^.

The wBL collected from the kraft pulping process contains about 15–20 wt% of dry solids, of which 2/3 is due to the presence of organic compounds while 1/3 is inorganic chemicals. To maximize energy production, the wBL is concentrated at about 65–75 wt%, reaching up to 80–85 wt% in the evaporator stage. Once the BL is concentrated, it is burned in the recovery boiler, which may be sufficient to provide steam and electricity to the mill^3–6^. However, recovery of inorganic chemicals from BL is necessary to maintain the cost-effectiveness of the pulping processes. It is estimated that 96–98% of alkaline chemicals can be recovered from kraft BL^1^. To attain the most favorable cost–benefit ratio and improve the integration of the pulp and papermaking process, other factors must be considered, such as decreasing the silicon content in BL and enlarging the capability of recovery systems within the mills^5^.

BL is a useful byproduct from the kraft pulping process because it contains a variety of valuable chemicals that can be recovered and reused. BL is a complex aqueous solution formed by dissolved organic compounds and inorganic alkali chemicals produced on wood kraft pulping^7^. The organic solids consist mainly of lignin degradation products (mostly high-molecular-mass fragments) and degraded cellulose and hemicelluloses (aliphatic carboxylic acids), in addition to a small fraction of extractives and other organic hemicellulose residues (oligo and polysaccharides)^8^. Inorganic components consist mainly of sodium salts and small amounts of potassium, calcium, magnesium, silicon, and iron salts^9^. The composition of BL strongly varies with raw material (wood species), unbleached pulp yield, composition and amount of white liquor applied (from the oxygen delignification stage), and Kappa number, as well as the digester conditions and the liquor-to-wood ratio^10,11^.

Its chemical composition impacts the physical properties, such as density, thermal conductivity, boiling point, heating value, and rheological properties of the BL. Wood is the main source of cellulosic fiber for pulp and paper production, with a consumption range between 2000 and 12,000 tons per day^12^. This fibrous material is mainly composed of lignin, hemicelluloses, and cellulose, in addition to minor amounts of low molecular weight compounds such as extractives and inorganics^13^. In general, softwoods, including pine, have lignin content around 25–32% and hemicellulose content around 30–32%. Hardwoods, such as eucalyptus, have lignin content between 18 and 25% and hemicellulose content between 15 and 35%. Cellulose is a relatively uniform component of both wood types (40–44%)^13^.

Black liquors have different characteristics depending on where they are processed and the type of wood they originate from. This work aims to conduct a comparative thermal and rheological study between Brazilian liquors from the hardwood of *Eucalyptus* sp., with Finnish liquor from the soft wood of *Pinus* sp.

### The black liquor

In terms of lignin constituents, softwood lignin consists of guaiacyl units, whereas hardwood lignin contains a mixture of guaiacyl and syringyl units^14^. Guaiacyl units have a free C–5 position that favors the formation of C–C bonds. Since guaiacyl-type lignin contains more resistant linkages that involve the C5 than the syringyl-type lignin, softwood lignin is more resistant to the delignification process and less reactive during the kraft pulping than hardwood lignin^15^. To maintain high yields and preserve a sufficiently high quality of the pulp, delignification is limited to a certain degree of lignin removal, targeting Kappa numbers of around 25–30 for softwood kraft pulps, and around 15–20 for hardwood kraft pulps^13^. Low Kappa numbers mean less residual lignin in the pulp and, therefore, a higher lignin concentration in the BL.

A comparative with literature on the molar mass of lignin for hardwood Eucalyptus BL and softwood *Pinus Caribaea* black liquor from Brazil and Scandinavia, respectively, found that the average lignin molar mass is lower for Eucalyptus liquors than for Pinus liquors. The authors suggest that the faster delignification process in hardwoods is responsible for their low lignin content. On the other hand, the high sodium content leads to an alkali environment that promotes the breakdown of the macromolecules^14–16^. These findings served as the basis to direct this work towards the study of the rheological properties of the BLs and the results are summarized in Table 1.

**Table 1:** Moisture content, dry solids content, and pH values for PBL and EBL^17^.

Black liquor	Origin	Kappa number	MM_lig_ (%)
Hardwood/*Eucalyptus grandis*	Brazil/Mill A	17	820
Hardwood/*Eucalyptus grandis*	Brazil/Mill B	17	1641
Hardwood/*Eucalyptus grandis*	Brazil/Mill C	17	1401
Hardwood/*Eucalyptus grandis*	Brazil/Mill D	17	1050
Hardwood/*Eucalyptus grandis*	Brazil/Mill E	17	1871
Softwood/*Pinus Caribaea*	Scandinavia	17–125	2728

*MM*_*lig*_ lignin molar mass.

BLs with high lignin concentrations tend to have a high viscosity because lignin can cluster into amorphous and voluminous molecules of high molar mass. However, BLs with low lignin content exhibit lower viscosity since lignin agglomerates in a compact and spherical molecular structure^7^.

Rheological properties allow the correlation of the transport behavior of BL with the efficiency capacity in the PPI. From the rheological standpoint, BL can be treated as a polymeric solution since lignin composes more than 50% of its organic constituents. Rheological properties of BL depend on wood type, lignin content, pulping type and conditions, dry solids content, and temperature^18^. Multiple researchers have conducted studies on the rheology of BLs derived from softwood, hardwood, and a mixture of both wood types^16,19–23^. Among the various rheological properties of BL, viscosity is of particular interest since highly viscous BLs are difficult to transport inside the pulp mill. Therefore, the study of this property is crucial to improve the pump design, transport through pipelines, recovery boiler spraying, and combustion efficiency.

The viscosity of BL is greatly affected by solids concentration as it increases exponentially with dry solids content. At low solids content, organic compounds are in a colloidal state, so the aqueous solution is the continuous phase. At high solids concentration, typically above 50%, the polymeric organic compounds become the continuous phase^24^.

This preliminary comparative study examines the thermal and rheological properties of BL samples obtained from two different types of wood: *Pinus* sp. from Finnish PPI and *Eucalyptus* sp. from Brazilian PPI. Other researchers have already reported the influence of molar mass distribution on the viscous response of BLs^17^; therefore, this study also aims to understand how temperature and shear deformation affect the apparent viscosity of the BLs, because a decrease in viscosity directly impacts the heat transfer coefficient and, thus, the evaporation capacity and heat transfer area during the BL combustion in the recovery boiler. Although many studies have been conducted on the rheological properties of BL from different types of wood, relatively few investigations have been performed on the dynamic shear properties of the liquors, which can provide insight into the elastic and viscous characteristics of BLs and their correlation with the conditions encountered in the pulp mill.

Liquors are classified as pseudoplastic. During shearing, this macrostructure may be restructured and tend to unfold in the flow direction, leading to decreased viscosity with increased shear rate.

**Figure 1:**
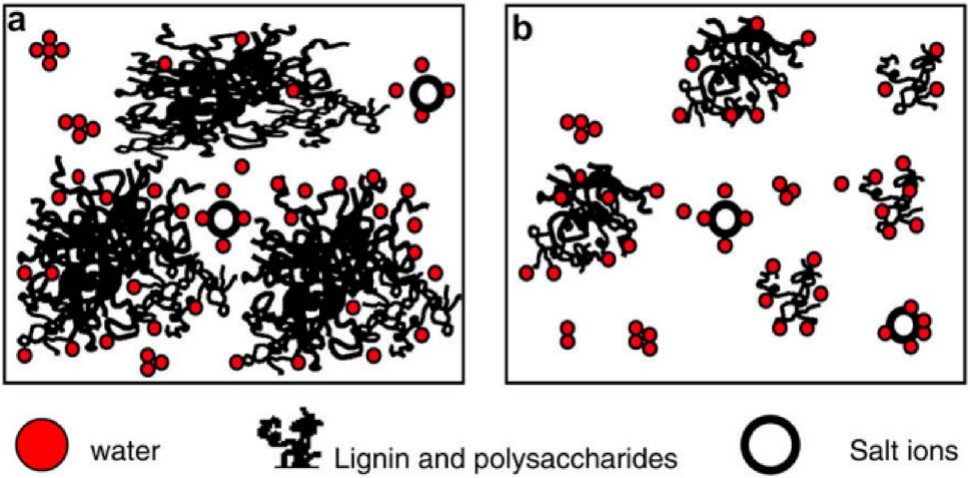
Schematic representation of lignin and polysaccharide conglomerates presented in black liquor: (**a**) voluminous and shapeless and (**b**) compact and spherical^7^.

The complex structure contains organic materials from wood or fibrous plants, resinous compounds with low molar mass, and inorganic compounds, mainly soluble saline ions. The typical folding of polymeric structures is represented in Fig. 1. Lignin works as a binding agent and is formed by phenyl-propane structures. During the pulping operation, lignin is fragmented, and carbohydrates are dissolved and converted into low molar mass acids, except for xylan (the main hemicellulose of hardwoods), which cannot be degraded and survives the pulping operation. Lignin and polysaccharides can assemble into bulky, amorphous structures with high molar mass, which are represented in Fig. 1a. On the other hand, structures with low concentrations of lignin and polysaccharides tend to have lower viscosity and present more compact and spherical structures, represented in Fig. 1b.

## Materials and methods

Finnish *Pinus* sp. black liquor (PBL) with 74.1 wt% ds and pH 13 was provided by a Finnish pulp and paper mill located in Lappeenranta city. Brazilian *Eucalyptus* sp. black liquor (EBL) with 80.8 wt% ds and pH 12.5 was provided by a Brazilian pulp mill located in the northeastern region of Brazil. Both PBL and EBL samples containing ashes were collected at the stage between the exit of the evaporation plant and the entrance of the recovery boiler. After collection, the BL samples were stored under refrigeration at 2 °C to avoid changes in their physical and chemical properties while slowing down the growth of bacteria and other microorganisms. Prior to the experimental analyses, the BL samples were removed from the refrigerator and allowed to come to room temperature for a period of 2 h.

### Dry solids content

Dry solids content can be expressed as the ratio between BL mass after thermal treatment and the initial mass of the raw sample^25^. For both liquors, it was calculated using TAPPI T650 om-21 standard, which consisted of drying a determined mass of each BL sample in an oven under controlled temperature (T = 105 ± 3 °C) until reaching a constant mass^26^. The results shown are the average of three measurements with their respective standard deviation for each BL sample.

### Fourier transform infrared spectroscopy on attenuated total reflectance (FTIR-ATR)

FTIR absorption spectra on ATR mode were obtained on a Perkin Elmer Frontier FTIR Spectrometer and on a Thermo Scientific Nicolet iS5 FTIR spectrometer for PBL and EBL, respectively. Both spectra were obtained between 4000 and 400 cm^−1^ at 4 cm^−1^ resolution. For sample preparation, a thin, uniform layer of BL samples was spread on the substrate of the equipment to ensure accurate and reliable results.

### Thermal analysis

To analyze the thermal events that occur in both liquor samples, thermogravimetric analysis (TGA) curves and differential thermogravimetric analysis (DTG) curves were obtained on a Netzsch STA 449C thermobalance for PBL and on TGA instruments TGA Q50 thermogravimetric analyzer for EBL. The temperature range was between 25 and 900 °C (298.15–1173.15 K) under Nitrogen atmosphere (40 ml min^−1^) and at a heating rate of 10 °C min^−1^. Approximately 10 mg of each BL was weighed and then placed on an Al_2_O_3_ crucible. To complement the TGA/DTG analyses, differential scanning calorimetry (DSC) curves were obtained on a Netzsch DSC 204 F1 Phoenix (PBL) and on a Perkin Elmer differential scanning calorimeter DSC 8000 (EBL) in the temperature range of 25–250 °C (298.15–523.15 K) at a heating rate of 10 °C min^−1^ and under Nitrogen atmosphere (40 ml min^−1^). Approximately 2 mg of BL was placed in an aluminum pan, and then the cell was sealed hermetically. The pan was hermetically sealed to avoid the diffusion of reactive gases and prevent oxidation processes. The latter condition is undesirable and is observed in realistic conditions, specifically in evaporator units in the pulp mill^25^.

### Rheology

Rheological properties of BLs at temperatures from 30 to 70 °C (303.15–343.15 K) were analyzed on an Anton Paar modular compact dynamic shear (DSR) rheometer. This temperature range was chosen to study the rheological properties of the BLs under moderate thermal conditions and below the glass transition temperature to guarantee the brittle, glassy state of the amorphous regions. For PBL, the MCR 302 model was used, and for EBL, the MCR 502. The geometries used were an 8 mm parallel plate (PP08) for PBL and a 15 mm parallel plate (PP15/TG) for EBL, both with a 1.0 mm gap.

The temperature was controlled using /TG+H-PTD200 Peltier system and a Hood accessory to avoid sample heat losses. Shear stress and viscosity curves of the BLs were obtained over a shear rate range of 0.001–100 s^−1^.

The rheological study makes it possible to parameterize the characteristics of the sample with the microstructure of the material, using the determination of the linear viscoelastic regime (LVE)^26,27^. The structural stability of BLs was studied with an angular frequency sweep between 100–0.1 rad s^−1^ with a constant shear strain of 0.1%. The choice of the shear strain value was to ensure that all the BL samples were within the LVE region^27^. The choice of test geometries generally meets the characteristics of the sample and the expected work range. However, the choice of these geometries for the analysis of the BLs in different laboratories, Finland and Brazil, was a function of the availability of these geometries in the analysis locations. Although the recommended geometries for BLs should be 8 mm, it is known that physically, the 8 mm and 15 mm geometries have a common range of shear overlap. This common range meets the demand of the black liquors analyzed, thus not causing an analytical problem for this preliminary assessment.

## Results and discussion

### Dry solids content analysis

The dry solids (ds) content, along with moisture content and pH values of both BLs, were calculated, and the results are listed in Table 2. As observed, the PBL has a lower ds content than the EBL. As discussed later in the TGA/DTG section, this may be associated with higher ashes and inorganics content in the Brazilian PPI liquor, since both BLs showed similar mass losses.

**Table 2:** Moisture content, dry solids content, and pH values for PBL and EBL.

Black liquor	Dry solids content (wt%)	Moisture content (%)	pH*
PBL	74.1 ± 1.4	25.9 ± 1.4	13.0
EBL	80.8 ± 1.8	19.2 ± 1.8	12.5

*Data provided by the forest industry companies.

### FTIR-ATR analysis

**Figure 2:**
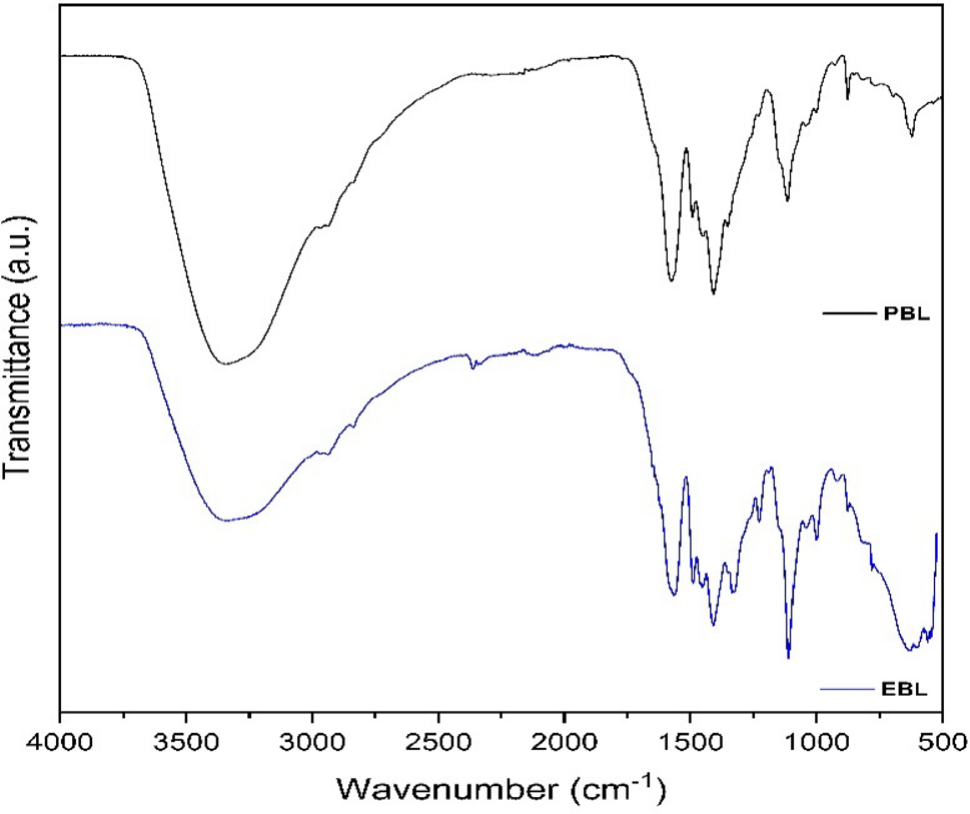
FTIR spectra on ATR mode for PBL (black line) and EBL (blue line).

The functional groups and chemical composition of PBL and EBL were investigated using FTIR-ATR analysis. Figure 2 shows both BLs FTIR spectra, and Table A1 in supplementary material lists the main vibrational frequencies and their corresponding assignments. Both spectra did not exhibit significant differences throughout the studied frequency interval, which allows us to affirm that both liquors have similar chemical composition. The characteristic absorption bands between 3342–3330 cm^−1^ are associated with O–H stretching vibrations due to the presence of phenol, alcohol, or carboxyl acid groups in lignin, in addition to water molecules^28^. The bands between 2970–2836 cm^−1^ are related to C–H stretching of methyl and methylene groups in lignin^29^. The bands between 1578–1563 cm^−1^ appear due to C–C stretching of aromatic rings present in polymeric macromolecules^29^, while the bands between 1492–1488 cm^−1^ are due to aromatic C = C stretching from guaiacyl lignin^30^.

Another absorption band was detected at 1451 cm^−1^ in both liquors. It is typically associated with the bending vibrations of the C–H bonds within the methyl (–CH_3_) and methylene (–CH_2_) groups in lignin^31^. The bands at 1408 and 1353 cm^−1^ are associated with symmetric COO^−^ stretching from carboxylate ions and C–O stretching vibrations in the lignin structure due to the presence of phenolic hydroxyl groups, respectively^30^. The region of the FTIR spectrum between 1140 and 1114 cm^−1^ is known as the lignin “S–H” region, as it contains peaks that correspond to the S–H stretching vibrations of the sulfonic acid groups present in lignin. This region is also referred to as the guaiacyl region, due to the presence of guaiacyl units in lignin polymer, which usually has a strong peak at 1122 cm^−1^^32,33^. The absorption bands between 1114–1110 cm^−1^ are characteristic of the deformation vibrations of the C–H bonds^34^, while the band at 1044 cm^−1^ reveals the presence of aliphatic OH or ether groups^29^. The band at 1003 cm^−1^ is associated with S–O stretching from sodium thiosulfate^30^, while the bands observed below 1000 cm^−1^ are characteristic of the deformation vibrations of C–H out of plane^28^. Supplementary material A describes the assignments of absorption bands and FTIR peaks for PBL and EBL.

### Thermal analysis

**Figure 3:**
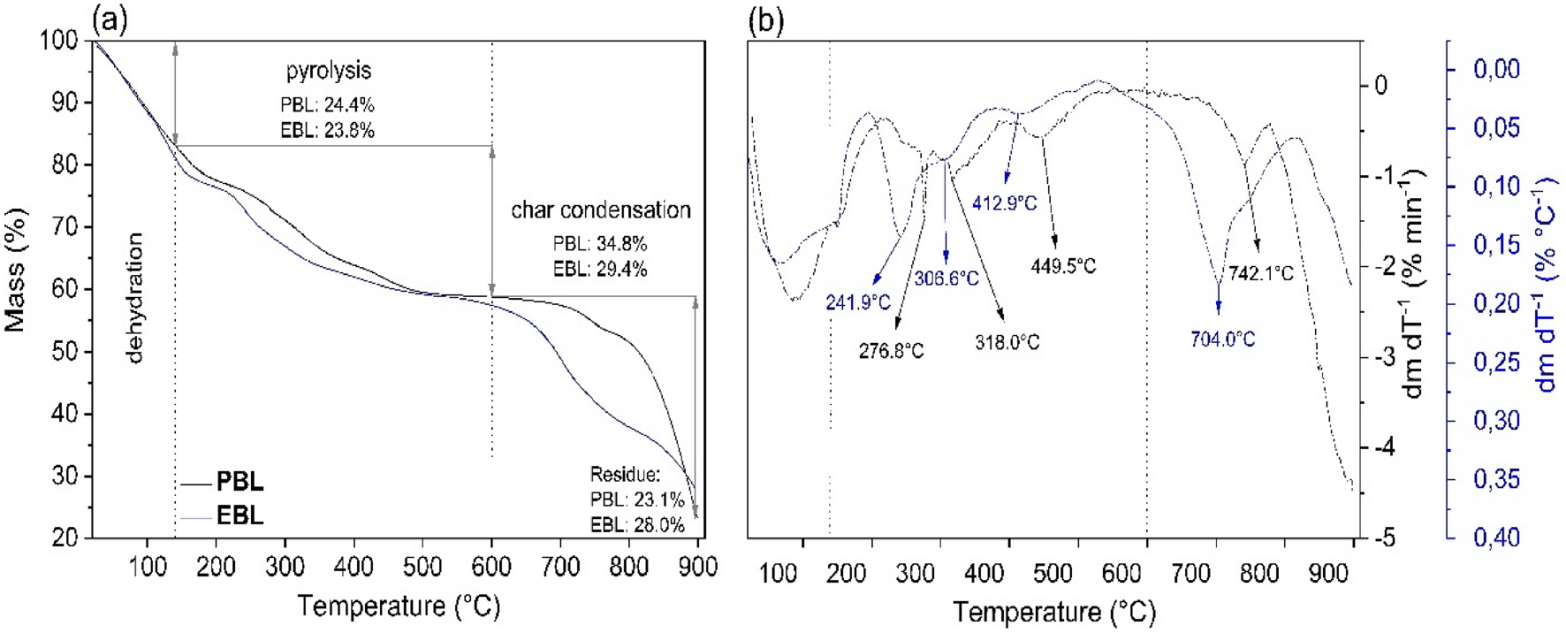
(**a**) TGA and (**b**) DTG curves for PBL (black lines) and EBL (blue lines).

The thermal properties of PBL and EBL were studied by TGA/DTG and DSC analysis. Table 3 lists the mass losses, thermal decomposition temperatures (T_d_), and glass transition temperatures (T_g_) for the samples studied. Figure 3 shows the mass loss curves as well as the rate of mass loss for both liquors. From the TGA curves in Fig. 3a, the different mass loss regions for each BL sample are related to different thermal degradation events. The diverse peaks in the DTG curves (Fig. 3b) expressed as T_d_, describe the thermal stability of the material^35^. The initial region between 25–140 °C shows mass losses of 16.8% (T_d_ = 85.2 °C) and 18.8% (T_d_ = 63.8 °C) for PBL and EBL, respectively, which are characteristic of water evaporation^25^. The observed difference in the water absorption can be attributed to two factors: one, PBL contains higher levels of lignin (between 25 and 32%) than EBL (between 18 and 25%)^13^, making it easier for water molecules to interact with the hardwood BL. Two, the presence of a higher residual mass in EBL due to the presence of ashes and metal ions leads to stronger electrostatic interactions between the water molecules and the liquor.

**Table 3:** Mass loss (%) and T_d_ (°C) values for PBL and EBL.

Black liquor	TGA/DTG							DSC
	Temperature range (°C)							
	25–140		140–600		600–900		Residual mass (%)	
	Mass loss (%)	T_d_ (°C)	Mass loss (%)	T_d_ (°C)	Mass loss (%)	T_d_ (°C)		*T*_*g*_ (°C)
PBL	16.8	85.2	24.4	276.8 318.0 449.5	34.8	742.1, 849.0	23.1	93.8
EBL	18.8	63.8	23.8	241.9 306.6 412.9	29.4	704.0, 849.0	28.0	97.2

A second large region between 140–600 °C with mass losses of 24.4% for PBL and 23.8% for EBL is associated with the pyrolysis stage of organic groups. In this temperature range, the thermal events occurring at 276.8, 318, and 449.5 °C for PBL and at 241.9, 306.6, and 412.9 °C for EBL, correspond to the thermal degradation of C_3_ branched chains, cleavage of ether bonds between aromatic monomers, and removal of methoxyl groups, respectively^28^. Although the mass losses are similar for both liquors, the thermal events for PBL occur at higher temperatures than those for EBL.

These results suggest that PBL offers greater resistance to thermal degradations than EBL. Since softwood (PBL) lignin consists mainly of guaiacyl units which favor the formation of C–C bonds, its three-dimensional structure will be more condensed and less reactive to thermal perturbations than hardwood (EBL) lignin, which is composed of various amounts of both guaiacyl and syringyl^36^.

### Rheological analysis

Rheological properties play a crucial role in the flow behavior of BL to transport, as well as conditions for its spraying through the nozzles and subsequent combustion in the recovery boiler unit. On the other hand, as the physical characteristics of BL have changed over time due to changes in its chemical composition and operating conditions of pulp mills, reporting the flow properties of liquors becomes important to improve and optimize evaporators and recovery furnace units. Because the exact composition of the studied BLs was not determined, the interpretation of their rheological properties has been primarily qualitative in relation to their composition and dry solids content.

### Steady shear properties of BLs

**Figure 4:**
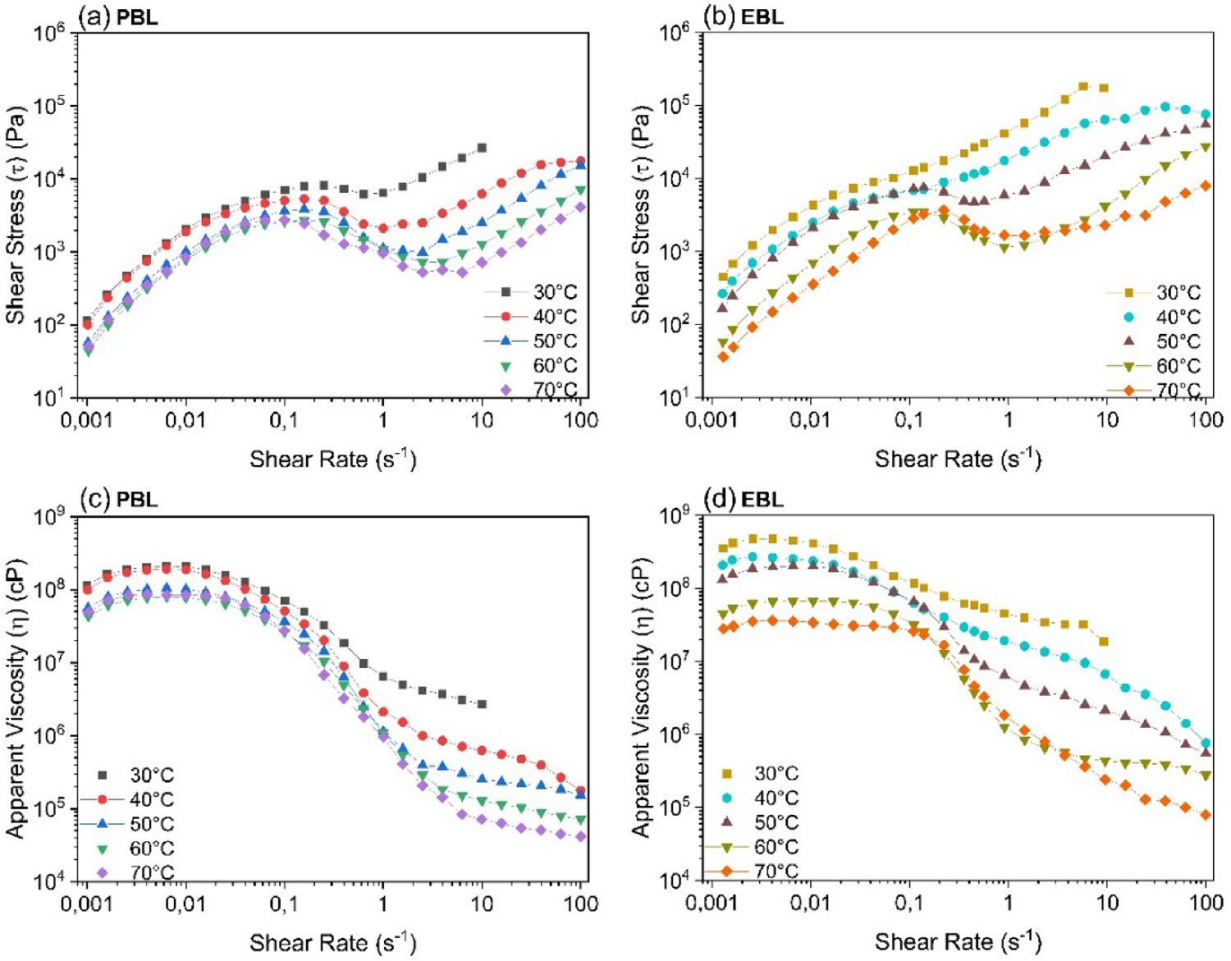
(**a**,**b**) Shear stress (τ) vs. shear rate curves and (**c**,**d**) apparent viscosity (η) vs. shear rate curves of (**a**,**c**) PBL and (**b**,**d**) EBL as a function of temperature.

Figure 4 shows the shear stress (τ) and apparent viscosity (η) curves as a function of shear rate ($\dot{{\varvec{\gamma}}})$ at temperatures from 30 to 70 °C. At low shear rates, the effect of increasing temperature is more pronounced in EBL than in PBL, given the variation of 55% for PBL and 92% for EBL.

This is in line with the results observed in the TGA/DTG curves. The lower thermal stability of EBL than PBL may be associated with a less rigid network structure. Measurements at low shear rates aim to understand the behavior pattern of BL for use in industry. As the shear rate increases, from 0.1 s^−1^, an inversion of the curve of tension occurs (Fig. 4a, b), which may be due to the displacements of the solids, until they assume the flow direction in the condition imposed by shear (~ 10 s^−1^), slowing down the reduction in viscosity (Fig. 4c, d). Industries should explore this very interesting effect for pumping BL.

Figure 4c, d show the effect of the temperature on the apparent viscosity of PBL and EBL, respectively. As expected, the η decreases as the temperature increases in both liquors. The decrease in the τ and η of black liquor with increasing temperature can also be explained by the presence of vacant lattice sites in liquids, which follows the Eyring theory^37^. As the temperature increases, the thermal energy causes the molecules to move more freely, allowing them to flow into these vacancies, and this flow requires energy^38^. As per Eyring relation, this flow is easier at higher temperatures, resulting in a decrease in shear stress and viscosity as the temperature increases.

Figure 4c, d show the characterization of additional rheological parameters of the BLs in relation to the shear rate. The slight increase in viscosity at low deformation rates (0.001 s^−1^) in both BLs is because the applied shear rate is insufficient to break the lignin molecular chain entanglements, allowing the polymeric molecules to interact more easily^38^. However, at 30 °C and 0.001 s^−1^, EBL has a viscosity 3.1 times higher than PBL. This trend is not observed at high temperatures, since at 70 °C, the viscosity of EBL is 43.6% lower than that of PBL. As the shear rate increases the viscosity of the BLs begins to decrease, thus exhibiting typical shear thinning behavior. This drop in viscosity, which is more pronounced at shear rates between 0.1–10 s^−1^, occurs due to the disentanglement of molecular chains in lignin, cellulose, and hemicelluloses, which do not resist the continuously applied shear force^5^. In addition, at 10 s^−1^, the viscosity of EBL is 7.0 times (30 °C) and 3.3 times (70 °C) higher than PBL. As the viscosity of the BLs is directly proportional to their ds content, an increase in this property is expected since EBL (80.8%) has a higher solids content than PBL (74.1%).

Finally, above 10 s^−1^, a slight transition from shear thinning to Newtonian behavior appears due to the breakdown of the polymeric compound networks, causing the BL polymer chains to align in the flow direction^38^. The results agree with those reported by Yue et al. (2017)^39^, who observed shear thinning behavior in bamboo kraft black liquor with solids content between 70.19 and 79.87 wt%, and a reduction in the liquor viscosity when the temperature increased from 70 to 98 °C.

Two other issues may be related to the increase in viscosity at low shear rates. First, the sample could present heterogeneity, with large particles out of step enough to cause this effect. The viscous and inertial factors would be responsible for this format. This is in line with the observations of Ewoldt, which could clarify this phenomenon^40^. The possibility of wall slippage was not observed during the tests, given the detailed instrumental programming.

The second issue is related to the works published by Mari et al., using mathematical simulation for non-monotonic flow curves in a suspension, and by Sánchez et al^41,42^. In the study of viscoelastic flow instability, who reported an S-shaped curve for non-monotonic flows in a certain volume fraction range and at low Reynolds numbers. The experiments by Mari et al. do not rule out the existence of finite macroscopic elasticity or even finite Brownian movement in flow curves at low shear rates. Combining elastic and inertial factors in fluids can cause certain instabilities, which was called “elastic-inertial turbulence” by Sánchez et al^42^. This could be explored in industrial practice to increase efficiency in mixtures with similar fractions at low Reynolds numbers^43^. In the experiments by Mari et al. literature, adjacent factors such as hysteresis were not studied for instrumental reasons; however, they addressed the existence of flow stability in uniform shear^41^. The issue of viscoelastic systems in non-monotonic flow is not a single problem but a sum of counterbalanced effects. These issues indicate the complexity of analyzing the viscosity of black liquor and must also be explored.

### Dynamic shear properties of BLs

**Figure 5:**
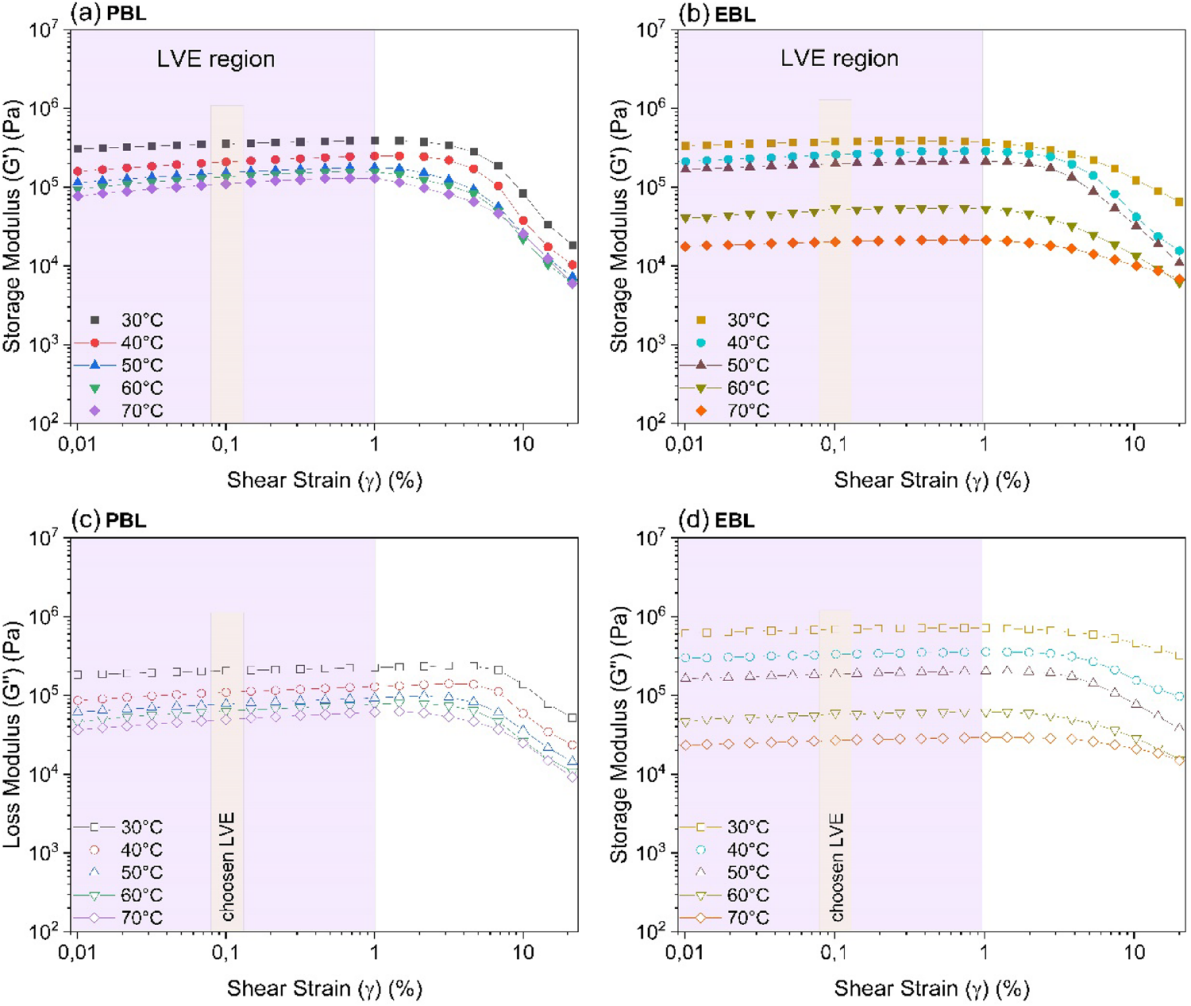
Dynamic strain sweeps for (**a**,**c**) PBL and (**b**,**d**) EBL as a function of temperature and at a constant frequency of 1 Hz. The solid symbols (**a**,**b**) represent the storage modulus (Gʹ), and the empty symbols (**c**,**d**) the loss modulus (Gʹʹ).

Storage modulus (Gʹ) describes the energy stored in a material during a shearing process. Loss modulus (Gʹʹ) describes the lost energy dissipated as heat or used for structural changes of the material^44^. Figure 5 shows the variation of Gʹ and Gʹʹ as a function of shear strain (γ) for PBL and EBL. The dynamic strain sweeps were performed between 30 and 70 °C and at a constant angular frequency (ω) of 1 Hz. The dynamic strain sweep is an important test as it provides information about the internal microstructure and viscoelastic contributions of the material. In the PBL curves, Gʹ (Fig. 5a) is greater than Gʹʹ (Fig. 5c) over a wide range of shear strain (0.01–6%), indicating that in PBL exhibits a liner viscoelasticity region^42^.

As shear strain increases, a crossing point where Gʹ = Gʹʹ appears indicates a transition from the elastic solid state to the liquid viscous fluid, which is typical of viscoelastic materials. This crossing point is temperature dependent as it gradually increases from 5.8% (30 °C) to 10.2% (70 °C). Contrary to what was observed in the PBL curves, Gʹ (Fig. 5b) is slightly smaller than Gʹʹ (Fig. 5d) for EBL over the entire range of shear strain. This means that EBL has a liquid-like property even at low oscillatory shear strains, thus displaying a viscous fluid behavior. When comparing the moduli of both liquors at low oscillatory deformations (0.01%), Gʹ and Gʹʹ in the EBL are 109.6% and 336.0% higher, respectively, than in the PBL at 30 °C. Nevertheless, when the temperature reaches 70 °C, EBL moduli decrease by 77.0% (Gʹ) and 35.8% (Gʹʹ) in relation to PBL.

The plateau region observed at low shear strains for both liquors extend up to 1%, as highlighted in the purple region of Fig. 5. This region is the indicated LVE regime, suggesting a microstructure of the polymeric compound networks in both BLs might recover their initial structural state once the oscillatory shear stress is removed^27^. However, liquors may present an amorphous structure, and this network is not easy to perceive. For treating liquors as polymers, future studies must be conducted to determine their degree of crystallinity and the level of crosslinked structures. Above 1%, Gʹ and Gʹʹ begin to decrease, suggesting that the critical strain deformation has been exceeded; thus, polymeric networks undergo irreversible structural changes due to the high applied shear strain^45^. When analyzing both moduli at γ_max_ (20%), the drop in viscoelasticity is less pronounced in EBL since at 30 °C the moduli are 2.8 times (Gʹ) and 15.7 times (Gʹʹ) higher compared to PBL, while at 70 °C the increase in the moduli magnitude is 1.5 times (Gʹ) and 2.2 times (Gʹʹ). This suggests that the EBL offers greater resistance to oscillatory deformation due to its high ds content (80.8%). Based on the considerations discussed above, a strain value within the LVE regime (γ_LVE_) of 0.1% was selected to perform the frequency sweep tests shown in Fig. 5.

**Figure 6:**
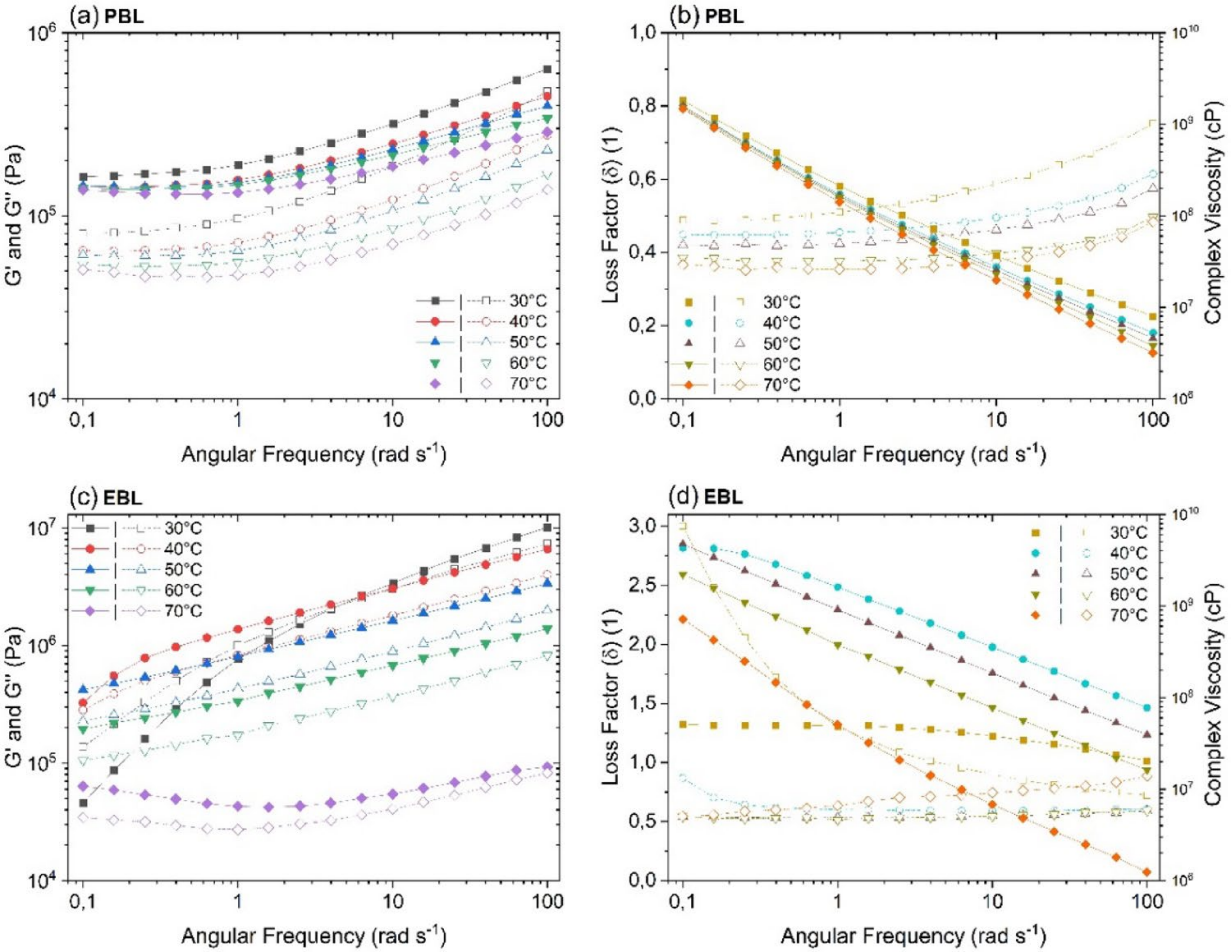
Dynamic frequency sweeps for (**a**,**b**) PBL and (**c**,**d**) EBL as a function of temperature. The solid and empty symbols in (**a**,**c**) represent the storage (Gʹ) and loss (Gʹʹ) moduli, respectively, while in (**b**,**d**) represent the complex viscosity and loss factor (δ), respectively. All the curves were obtained at a constant shear strain of 0.1%.

Figure 6 presents the variation of G*ʹ* and G*ʹʹ* together with the loss factor (δ) and complex viscosity (η*) as a function of angular frequency for PBL and EBL. The dynamic frequency sweeps were performed between 30 and 70 °C and at a constant shear strain, γ_LVE_, of 0.1%. The loss factor (or loss tangent) can be defined as the ratio between the loss modulus and the storage modulus, i.e., tan(δ) = G*ʹʹ*/G*ʹ*. In an elastic solid, tan(δ) = 0, meaning that G*ʹ* dominates over G*ʹʹ*. However, in a viscous fluid, tan(δ) = ∞, which means that G*ʹʹ* dominates over G*ʹ*. Viscoelastic materials usually 0 ≤ tan(δ) ≤ ∞ depending on the time scale and temperature. When G*ʹ* = G*ʹʹ*, i.e., tan(δ) = 1, the material is making a transition from liquid to solid state or vice versa^44^.

G*ʹ* > G*ʹʹ* for PBL throughout the studied frequency domain. This indicates that PBL shows a well-defined solid-like characteristic. This strong viscoelastic behavior is typical of an elastic material and means that the recovery of the deformation is greater than the irreversible deformation^5^. The stable plateau region observed between 0.1 and 1 rad s^−1^ indicates that the polymeric compound networks in PBL were not broken under low oscillatory deformations^45^. Above 1 rad s^−1^, both moduli become frequency-dependent as they increase with increasing frequency. The loss factor curves for the PBL in Fig. 6b reveal that at ω_max_ (100 rad s^−1^), tan(δ) ranges from 36.9° (30 °C) to 25.8° (70 °C), confirming the highly crosslinked network structure of the lignin polymers present in the Finnish liquor. Furthermore, over the entire range of oscillating shear frequency, η* decreases by three orders of magnitude with increasing ω, which is analogous to the steady shear regime (Fig. 6) and reveals the shear thinning behavior of the *Pinus* sp. BL in the oscillating shear field.

From a structural point of view, the dominant solid-like behavior, which is more evident in PBL, means that a greater yield stress would be required to break the high-strength polymeric networks^18^. However, droplet size and size distribution are the most important parameters to achieve high combustion efficiency of BL in a pulp and mill recovery boiler. The BL droplets must be small enough to dry before reaching the char bed and large enough to avoid being entrained in the furnace gas flow^22^.

Under realistic flowing conditions in the pulp mill, *i.e.*, mass flows between 1000 and 4000 s^−1^ and temperature around 120 °C, a decrease in the viscoelasticity of BL is desirable as it makes the liquor softer, facilitating its flow through pipes and atomization through nozzles while maintaining the polymer structure. The decrease in viscoelastic behavior with increasing temperature for the concentrated BLs (74.1 wt% for PBL and 80.8 wt% for EBL) suggests that there will be no issues during the droplet formation and combustion in the recovery furnace. In addition to temperature, an increase in angular frequency also decreases the degree of viscoelasticity, as observed in Table 4. The ratio of storage viscosity (${\eta }^{{\prime}{\prime}}=G{\prime}/\omega$) to dynamic viscosity (${\eta }{\prime}=G{\prime}{\prime}/\omega$), which relate the energy storage and viscous contribution of the fluid, respectively, infers that as angular frequency increases in both liquors, the ratio of $\eta {\prime}{\prime}/\eta {\prime}$ decreases at 70 °C.

**Table 4:** Dynamic ($\eta {\prime}$) and storage ($\eta {\prime}{\prime}$) viscosities for PBL and EBL obtained at 70 °C and different angular frequencies.

Liquor	Dry solids content (wt%)	$\eta {\prime}{\prime}/\eta {\prime}$ at $\omega =0.1\,{rad\,s}^{-1}$	$\eta {\prime}{\prime}/\eta {\prime}$ at $\omega =1\,{rad\,s}^{-1}$	$\eta {\prime}{\prime}/\eta {\prime}$ at $\omega =100\,{rad\,s}^{-1}$
PBL	74.1	2.7213	2.8254	2.0672
EBL	80.8	1.8359	1.5757	1.1272

The rheological results in the dynamic shear regime are in agreement with the findings of Zaman and Fricke (1995) in pine kraft BL (75.7–81.1 wt% solids), Xu et al. (2016) in bamboo kraft BL (63.1–77.4 wt% solids), and Choi et al. (2019) in micro fibrillated cellulose fluids (Fig. 5).

## Conclusions

The structural, thermal, and rheological properties of softwood—*Pinus* sp. (74.1 wt% ds) and hardwood—*Eucalyptus* sp. (80.8 wt% ds) BLs from a Finnish pulp and paper mill and Brazilian pulp mill, respectively, were studied.

FITR measurements confirmed the presence of lignin, hemicelluloses as well as functional groups in both liquors. TGA/DTG curves showed three stages of thermal decomposition associated with water evaporation, pyrolysis of organics groups, and char condensation. The thermal degradation temperatures in the pyrolysis stage were higher for PBL due to the high density of guaiacyl units, which indicates a more condensed PBL structure and less reaction to thermal perturbation. The residue of the TGA/DTG analysis was higher for the EBL (28.0%) compared to the PBL (23.1%), which suggested a higher amount of inorganics, ashes, and residual carbon in the Brazilian liquor samples.

The Liqueurs present a variation in behavior that characterizes them as non-Newtonian and having pseudoplastic behavior, even though, at low shear rates, there may be a constant linear relationship between tension and shear rate and, the inflections of the curves. When comparing the viscosities of the two types of liquors at a shear rate of 0.001 s^−1^, EBL had a 3.1 times higher viscosity at 30 °C and 43.6% lower viscosity at 70 °C than PBL. The observations on the “S” curves discussed in Fig. 4, and the effects on viscoelasticity at low shear rates draw attention to a behavior that can be observed in liquors. This behavior could be very interesting to explore in the composition of mixtures at low Reynolds numbers.

The effect of oscillatory deformation on the flux properties of the liquors beyond the LVE domain limit (up to 1%) and the Gʹ and Gʹʹ curves for both types deviated from a constant plateau and decreased, indicating irreversible structural deformation. The frequency sweeps showed that Gʹ > Gʹʹ for PBL throughout the studied frequency domain and at all temperatures, confirming the strong solid-like characteristic of the PBL. When comparing the viscoelastic nature of both liquors, the solid-like behavior was more pronounced and well-defined in PBL, probably due to the presence of more guaiacyl units in its structure. However, with increasing temperature and angular frequency, both liquors showed a drop in dynamic shear properties, which indicates that at high temperatures and oscillatory deformations, there will be no issues during droplet formation and size distribution; therefore, high combustion efficiencies can be achieved in the recovery furnace.

### Supplementary Information

Supplementary Information.

## Data Availability

All data generated or analyzed during this study are included in this published article and its supplementary information file.
