# Supplementary material for: Comparison of thermal, rheological properties of Finnish Pinus sp. and Brazilian Eucalyptus sp. black liquors and their impact on recovery units
Source: Sci Rep. 2024 Jul 5;14:15498. doi: 10.1038/s41598-024-66513-z (PMC11226715; doi:10.1038/s41598-024-66513-z)
Supplement: Supplementary file 1 — Supplementary Information. [file 41598_2024_66513_MOESM1_ESM.docx]

**Supplementary material A**

**Comparison of thermal, rheological properties of Finnish *Pinus sp.* and Brazilian *Eucalyptus sp.* black liquors and their impact on recovery units**

Jesús Nuncira^a^, Getúlio Francisco Manoel^b^, Larisse Ap. Ribas Batalha^c^, Lindomar Matias Gonçalves^d^, Clara Mendoza-Martinez^a,*^, Marcelo Cardoso^e^, Esa K. Vakkilainen^a^.

^a^ LUT University, Yliopistonkatu 34, FI-53850, Lappeenranta, Finland

^b^ Pontifical Catholic University of Minas Gerais (PUC Minas), Belo Horizonte, MG, 30535-000, Brazil

^c^ Federal Rural University of Rio de Janeiro (UFRRJ), Seropédica, RJ, 23890-000, Brazil

^d^ Federal University of Itajubá (UNIFEI), Institute of Pure and Applied Sciences, Rua Irmã Ivone Drumond, 200 - Industrial District II, MG 35903-087 Itabira, Brazil.

^e^ Federal University of Minas Gerais (UFMG), Belo Horizonte, MG, 31270-901, Brazil

^*^ Corresponding author: E-mail address: [clara.mendoza.martinez@lut.fi](mailto:clara.mendoza.martinez@lut.fi)

FTIR absorption bands and peaks assignments for *Pinus sp.* (PBL) and *Eucalyptus sp.* (EBL)

**Table A1:** FTIR absorption bands and peaks assignments for PBL and EBL.

| Wavenumber (cm^-1^) | Assignments | Ref. |
| --- | --- | --- |
| 3342 – 3330 | O–H stretching of phenols, alcohols or carboxyl acids groups in lignin | ^29^ |
| 2970 – 2836 | C–H stretching of methyl and methylene groups in lignin | ^30^ |
| 1578 – 1563 | C–C stretching of aromatic rings of polymeric macromolecules | ^30^ |
| 1492 – 1488 | aromatic C=C stretching from guaiacyl lignin | ^31^ |
| 1451 | C–H deformation of asymmetric -CH3 and -CH2 in lignin | ^32^ |
| 1408 | symmetric COO^-^ stretching from carboxylate ions | ^31^ |
| 1353 | C–O stretching vibrations in the lignin structure | ^31^ |
| 1140 – 1114 | S-H stretching vibrations of the sulfonic acid groups present in lignin | ^33,34^ |
| 1114 – 1110 | deformation vibrations of the C–H bonds | ^35^ |
| 1044 | aliphatic OH or ether groups | ^30^ |
| 1003 | S–O stretching from sodium thiosulfate | ^31^ |
| below 1000 | deformation vibrations of C–H out of plane | ^29^ |
